# Among-population divergence in personality is linked to altitude in plateau pikas (*Ochotona curzoniae***)**

**DOI:** 10.1186/s12983-019-0329-6

**Published:** 2019-07-08

**Authors:** Jiapeng Qu, Denis Réale, Quinn E. Fletcher, Yanming Zhang

**Affiliations:** 1 Key laboratory of adaptation and evolution of plateau biota, Northwest Institute of Plateau Biology, Chinese Academy of Sciences, Qinghai, 810008 China; 2 Département des Sciences Biologiques, Université du Québec à Montréal, Montréal, QC H3P 3P8 Canada; 3 Department of Biology and Centre for Forest Interdisciplinary Research (C-FIR), University of Winnipeg, Winnipeg, MB R3B 2E9 Canada; 4 State Key Laboratory of Grassland Agro-ecosystems SKLGAE, Lanzhou University, Lanzhou, 730000 China; 5 Qinghai Provincial Key Laboratory of Animal Ecological Genomics, Qinghai, 810008 China

**Keywords:** Altitude, Life history, Pace-of-life syndrome, Personality

## Abstract

**Background:**

Animals inhabiting high altitudes consistently show slow life-histories. The pace-of-life syndrome (POLS) hypothesis posits behavioural, physiological and/or morphological traits that mediate the trade-off between current and future reproduction or survival, which have coevolved along a slow-fast life history continuum. Previous studies have shown that the life histories of plateau pikas varied across altitude, high-altitude individuals showed slow pace of life which were characterized by few litters per year with small litter sizes. Thus, we hypothesized that pikas populations at higher altitudes would also express personalities characteristic associated with slow life history, such as high sociability, low activity or aggressiveness. We tested this hypothesis by comparing the activity and docility of three plateau pika (*Ochotona curzoniae*) populations distributed along an altitudinal gradient of the Tibetan Plateau. We predicted that high-altitude pika would be more docile and less active.

**Results:**

The behaviour of 556 pikas, from which 120 individuals were measured at least twice, was quantified. We observed that plateau pikas at high altitudes were less active and more docile than pika at lower altitudes. Activity and docility were significantly and negatively correlated in populations from high altitudes but not in populations from low altitudes.

**Conclusions:**

Our results support the POLS hypothesis, highlight the existence of personality variation among populations distributed along an altitudinal gradient and emphasise the importance of environmental selection on personality divergence.

## Background

Species with wide ranges usually exhibit geographic variation in life history traits, such as growth and reproduction [1–3]. Geographic variation in life history traits is becoming an important avenue for research in ecology and evolutionary biology [4, 5]. Geographic variation in life history traits arises from intrinsic factors (i.e. genetic and/or genomic divergence amongst populations) or extrinsic factors (e.g. temperature, precipitation, food availability and predation) [6–8]. Selection operates in different directions and/or with differing strengths across the geographic range of species, resulting in distinct life history strategies contributing to local adaptation.

Populations inhabiting altitudinal gradients experience distinct environmental conditions and show divergent traits in response to these conditions. With an increase in altitude, ambient temperature and growing season duration and biomass decrease, and plant phenology is delayed [9]. Specific species with wide altitudinal ranges adopt altitude-specific life-history strategies, commonly showing slower life histories with increasing altitude [9, 10]. For example, dark-eyed Juncos (*Junco hyemalis*) bred at high elevations showed delayed development of structures necessary for reproduction, reduced duration of reproductive period and fewer broods than low-elevation conspecifics [10, 11]. Moreover, amphibians and mammals at higher elevations exhibit shorter breeding seasons, longer larval periods and fewer litters per year with lower litter sizes compared with individuals at lower elevations [2, 12].

The pace-of-life syndrome (POLS) hypothesis posits that any trait (behavioural, physiological or morphological) that mediates trade-off between current and future fitness should coevolve with the life-history traits along the slow-fast life-history continuum [13–15]. The narrow-sense version of the POLS hypothesis [14] predicts that a fast pace of life, which is characterized by a short lifespan, an early age of first reproduction and a fast growth rate, is associated with a more bold, active and aggressive personality type that explores the environment in a cursory manner [16–18]. Individuals inhabiting populations exposed to differing environmental conditions are assumed to show different pace-of-life types [19–23]. For example, blue tits (*Cyanistes caeruleus*) living in habitats that are either resource rich or poor show divergence in their life histories, activity and docility following the predictions of the POLS hypothesis [24, 25]. Ant (*Temnothorax longispinosus*) colonies from warmer areas are more exploratory and less aggressive than ants from colder areas [26]. Although numerous studies have described altitude-associated variation in life histories [11, 27], no study has examined personality variation along an altitudinal gradient. A limited number of studies have also analysed personality divergence among populations living in different habitats [24, 26].

Here, we test the hypothesis that the mean level of personality change with altitude by comparing activity and docility of three plateau pika (*Ochotona curzoniae*) populations distributed along an altitudinal gradient [28]. Although the altitude difference between highest and lowest sites is about 700 m, annual and maximum monthly average temperature and vegetation conditions show distinct differences among sites (Table 1). Earlier research has shown that pika populations manifest slower life-histories at higher altitudes characterized by fewer litters per year with lower litter sizes and a shorter breeding season compared with populations at lower altitudes [29–34]. We predicted that high-altitude populations would be characterised by slower individuals, which are characterized by more thorough activity and lower docility, than high-altitude populations [13].

**Table 1:** Geographic, climatic, vegetation and pika life history characteristics of three sites in the eastern Tibetan Plateau (China)

Traits		Populations/ sites		
		Maqin (MQ)	Zeku (ZK)	Guinan (GN)
Geography^a^	Latitude (N)	34°24′	35°01′	35°28′
	Longitude (E)	100°21′	101°27′	101°08′
	Altitude (m)	3984	3694	3307
Climate^b^	Annual precipitation (mm)	528	477.48	407.8
	Annual avg. temperature (°C)	−0.19	0.41	2.3
	Min. monthly avg. temperature (°C)	−12.6	−12.3	−11.5
	Max. monthly avg. temperature (°C)	9.7	6.74	13.2
	Annual sunshine duration (hs)	2260	2620.47	2701
Vegetation^a^	Cover^c^ (%) (mean ± SE)	75.2 ± 2.87	78.8 ± 3.05	85.8 ± 3.66
	Height^c^ (cm) (mean ± SE)	3.06 ± 0.23	3.2 ± 0.12	4.0 ± 0.19
	Growing season (days)	90–110	100–110	120–130
Life history	Breeding season	Apr.–Jun.^[28]^	Apr.–Jun.^a^	Apr.–Jul.^[22, 23, 30]^
	Litters per year	1–2	1–2	2–3
	Litter size (Mean ± SE)	3.2 ± 0.1^[22]^	4.4 ± 0.3^a^	4.5 ± 0.1^[19]^
	Annual survival rate (%)	33.5–50.3^[22]^	–	23.4–28.6^[30]^

^a^ data in present study, ^b^ climatic data obtained from the Qinghai Meteorological Bureau

^c^ vegetation cover and heights in GN were significantly higher when those in ZK and MQ (all *p* < 0.05)

## Methods

### Study system

The plateau pika is a small, diurnal lagomorph that is widely distributed throughout the alpine meadows of the Tibetan Plateau. Populations can live between altitudes of 3,200 and 5,300 m [35]. Pikas live communally forming territorial, mixed-sex groups that use a distinct burrow system with multiple entries [33]. Previous studies have shown population divergence in life history traits at different altitudes [29–33, 36–41]. For example, low-altitude populations (e.g. Guinan, 3,200 m) produce 3–5 litters per year with 4.5 ± 0.1 pups in each litter [31, 37], whereas those from high-altitude (e.g. Guoluo, 4,000 m) produce 1–2 litters per year with 3.2 ± 0.1 pups in each litter [32, 34].

**Fig. 1:**
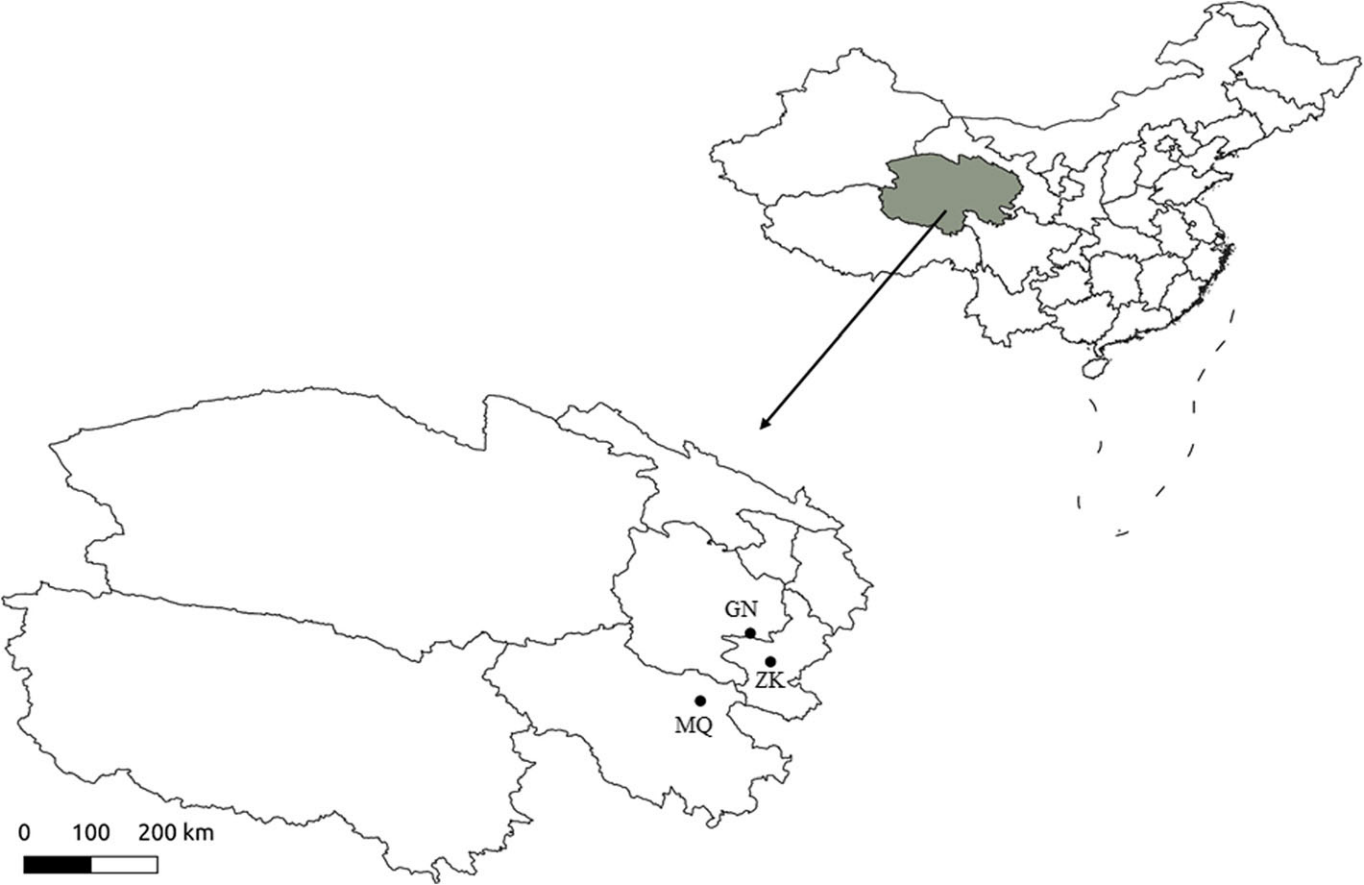
Map showing the locations of the study sites indicated by abbreviations corresponding to Table 1

We investigated three plateau pika populations at three different altitudes: Maqin (MQ), Zeku (ZK) and Guinan (GN), in the eastern Tibetan Plateau, Qinghai, China (Fig. 1). The distances between sites ranged from 180 km to 285 km, and the normal dispersal distance of plateau pika is about 40 m [42]; therefore, gene flow among the three populations is limited. At all sites, the climate is dry and cool, typical of a plateau continental climate [43]. Precipitation occurs mostly from May to September, and heavy winter snowfalls are rare. Daily temperatures vary considerably (up to 25 °C). The climate is harsher at higher altitudes with decreasing annual temperatures, whereas annual precipitation increases. As a result, productivity and vegetation height decreases, and the growing season becomes shorter at higher altitudes (Table 1).

Vegetation at the three sites is typical of Tibetan alpine meadows. The dominant plant species include *Kobresia humilis*, *Elymus nutans* Griseb. and *Leontopodium alpinum*. To evaluate vegetation at each site, we quantified plant cover and height using the approach developed by Huenneke et al. [44]. At each site, 10 quadrats (50 cm × 50 cm) were randomly selected. We estimated plant biomass within each quadrat using non-destructive measurements of plant species cover and height and total vegetation cover by counting the portions of squares occupied by plants. Vegetation height was presented as the mean height of each species. With the increase in altitude, vegetation cover and height were significantly higher at GN than at GL and ZK (Table 1).

At all sites, grass is grazed all year round by livestock (i.e. yak, sheep and horse). Other small herbivores, such as woolly hares (*Lepus oiostolus*), Himalayan marmots (*Marmota himalayana*) and plateau zokors (*Myospalax baileyi*), were absent at the sites. Common predators include red fox (*Vulpes vulpes*), Tibetan fox (*V. ferrilata*), upland buzzard (*Buteo hemilasius*) and saker falcon (*Falco cherrug*).

### Animal marking

The study area at each site measured 1.0 ha (100 m × 100 m). The study period was divided into two stages: breeding (June) and nonbreeding season (August). We caught pikas using string nooses, which were anchored into the soil with chopsticks (i.e. a chopstick with a loop of string with a slipknot that tightens around the neck of the pika), set up at multiple entries to the burrow system. This method is widely used in live-trapping of both lizards and mammals and causes no significant negative effects on the behaviour of animals [28, 29, 45]. Over 2005 to 2018 of research at these sites, no pikas have been accidently injured nor killed by the string nooses [28, 33, 34]. Live traps were set at 07:00–09:00, constantly observed from 30 m away by a field technician, and retrieved at approximately 13:00. Trapped individuals were extracted from the nooses within approximately 30 s after capture. Pikas were weighed, and their sex and reproductive status (active or inactive) was examined by checking their reproductive organs. We then determined the approximate age of each individual based on their body mass, fur colour and toe hardness [33]. Individuals were divided into two age classes: adults (born during the previous year) and juveniles (born during the year of the study). Individuals were marked using hand-made aluminium ear tags stamped with a unique number code for permanent identification (ID). All individuals were released on the study grid where they were captured.

### Behavioural tests

We conducted behavioural assays on recapture events on different days following the first capture when individuals were marked. After capturing, a field technician immediately transported the pikas to a processing location located ~ 30 m off the trapping grid. Animals were transported in mash bags, and ~ 1 min was needed to reach the processing location. At the processing location, the behaviour of the pika was immediately examined according to the following sequence:

(1) Bag test: The pika was transferred into a mesh bag (20 cm × 40 cm). We used the number of seconds the pika remained immobile during 1 min as an index of docility [46].

(2) Open field test: Pikas were transferred from the mesh bag into an open field arena. The arena was a wood box (100 cm × 100 cm × 100 cm), painted white and with grid (10 cm × 10 cm) lines drawn on the floor. We recorded open field trails for 120 s using a digital video camera fixed on a tripod. The pikas were then removed from the arena, and their ID were recorded. Pikas were then released at their location of capture. After each trial, the arena was cleaned using 75% alcohol and air-dried prior to the subsequent trial. Using EthoVision XT 9.0 (Noldus Information Technology), we measured the distance covered by each pika (cm) as an index of activity [17, 47]. Individuals from MQ were tested in June (*n* = 165) and August (*n* = 205) 2013, individuals from GN were tested in June 2013 (*n* = 17) and August 2014 (*n* = 31), whereas those from ZK were tested in June (*n* = 72) and August (*n* = 66) 2014. The interval time between repeated tests range from 75 to 104 days in three populations.

### Statistical analyses

Overall, the behaviour of 556 pikas, from which 120 individuals were measured twice, was quantified. Univariate linear mixed models (LMM) were used to test for phenotypic differences among populations and to estimate the repeatability of two traits. All univariate LMMs analyses were conducted using the R package MCMCglmm [48, 49]. For each model, we included population, sex, season (breeding versus nonbreeding) and age (juvenile versus adult) as fixed effects in addition to the random effects of ID and test date. We uses an inverse-Gamma distribution as the prior for variance components, the set of parameters were nu = 1.002, and V = 2 [49]. Models were run for 6,500,000 iterations with a thinning interval of 5,000 and a burn-in of 1,500,000. We determined the effects of traits based on whether their 95% credible intervals (CIs) overlapped with zero. We estimated repeatability and their 95% CIs following the approach of Dingemanse et al. [48]. Studies have proven that within-individual correlations were negligible, and phenotypic correlations can represent among-individual correlations in plateau pika population [28], thus, we tested the phenotypic correlations in this study. Pearson’s correlation tests were used when the original variables fitted a normal distribution or when log or square root transformation resulted in normality (Shapiro–Wilk test). Otherwise, Spearman’s rank correlation test was used. All analyses were carried out using *R* [50].

## Results

Activity and docility were highly (*r* = 0.77, CI = [0.70, 0.81]) and moderately (*r* = 0.46, CI = [0.37, 0.55]) repeatable, respectively, indicating that variation in these two personality traits varies consistently among individuals over time.

**Fig. 2:**
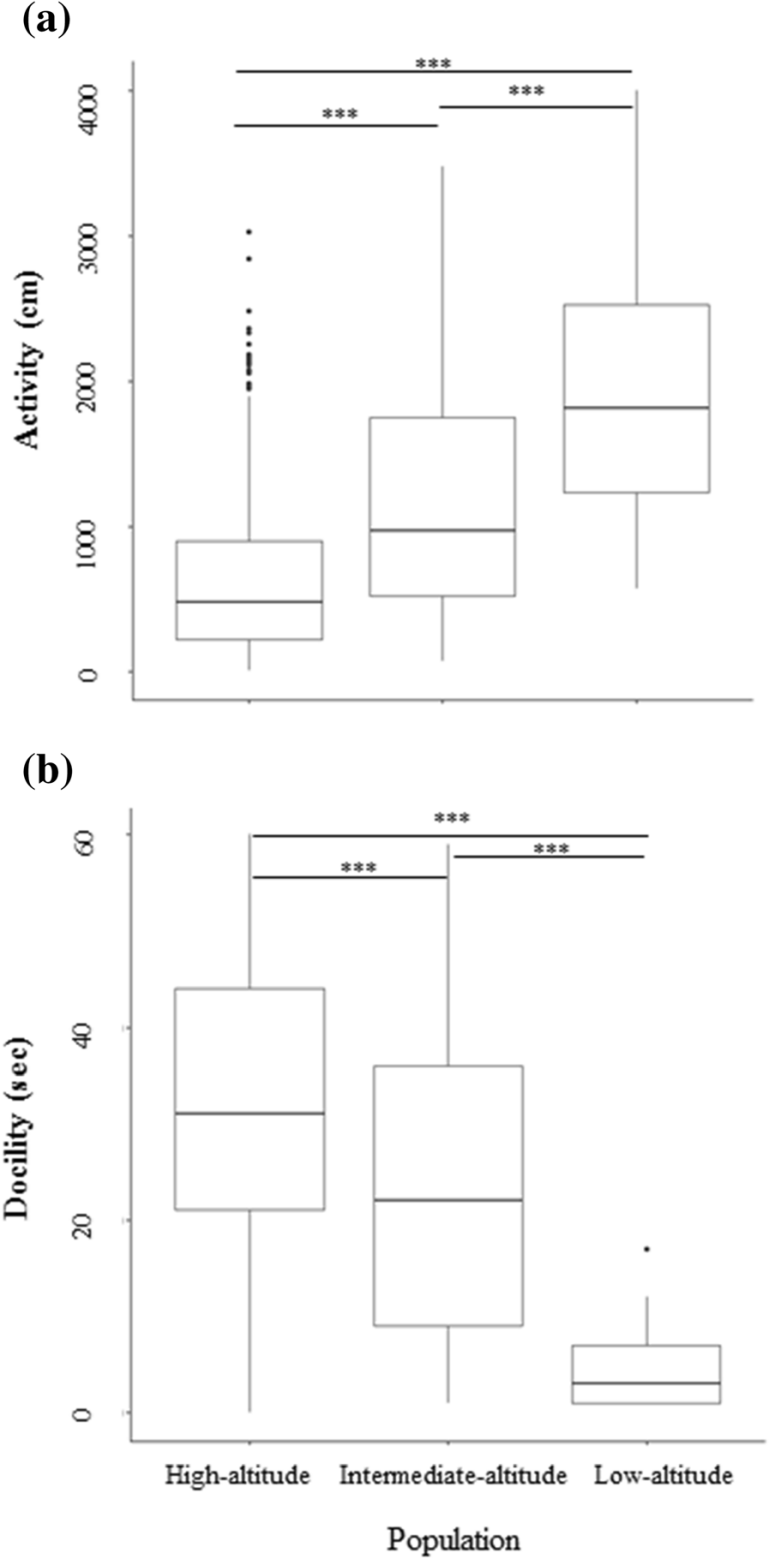
Activity (**a**) and docility (**b**) of plateau pika individuals inhabiting populations at three altitudes in the Tibetan Plateau. “^***^” indicates a significant difference (*P* < 0.001) between two populations

Three populations at different altitudes showed our predicted differences in activity and docility (Table 2). Activity of high-altitude individuals (MQ) was lower than in intermediate-altitude (ZK) and low-altitude individuals (GN) (Fig. 2a). High-altitude individuals were the most docile, followed by individuals at intermediate and low altitudes (Fig. 2b). Activity was significantly negatively correlated with docility in the MQ (*r* = − 0.172, *P* < 0.001) and ZK (*r* = − 0.432, *P* < 0.001) populations but showed no correlation with docility in the GN (*r* = − 0.286, *P* = 0.118) population.

**Table 2:** Univariate mixed models describing inter-population differences in personality traits across three plateau pika populations from the eastern Tibetan Plateau

Trait	Fixed effects	Estimate (CI 95%)	*Pmcmc*
Activity	Intercept	23.97 (22.03, 26.11)	**< 0.001**
	Population (ZK)	7.85 (4.76, 10.81)	**< 0.001**
	Population (GN)	19.66 (15.28, 23.75)	**< 0.001**
	Year	4.44 (−89.11, 91.88)	0.960
	Sex (M)	−11.44 (−-127.60, 104.10)	0.836
	Season (NB)	−1.06 (−3.29, 1.35)	0.158
	Age (J)	−0.05 (−2.23, 2.37)	0.792
	Population (ZK) × Season (NB)	2.12 (−1.07, 5.50)	0.21
Docility	Intercept	38.88 (36.61, 41.38)	**< 0.001**
	Population (ZK)	−9.09 (−13.54, −5.08)	**< 0.001**
	Population (GN)	−18.36 (−24.95, − 11.54)	**< 0.001**
	Year	−8.67 (− 18.20, 0.66)	0.080
	Sex (M)	−2.41 (−4.85, 0.23)	0.078
	Season (NB)	−2.96 (−6.37, 0.26)	0.096
	Age (J)	−3.14 (−6.08, 0.28)	0.064
	Population (ZK) × Season (NB)	−3.72 (−9.12, 1.76)	0.178

Bold values indicate significant effect

## Discussion

We investigated the phenotypic divergence of personality traits among plateau pika populations along an altitudinal gradient. Previous work on plateau pika populations indicates that the life-histories of high-altitude populations are slow. In support of the POLS hypothesis, we observed that higher-altitude populations also possessed personality traits that have been characterized as slow, i.e., less active and more docile, compared with the lower-altitude populations.

The differences in activity and docility among plateau pika altitude-associated populations can be attributed to the differing life history strategies that have evolved in response to different climates and environments (Table 1). Low-altitude pikas from GN show high reproductive output, which may be correlated with behaviour associated with increased energy intake [13, 51]. Activity in the open field is associated with activity levels, which are required for foraging, mating and predator escape [52]. Docility is often used as a measure of risky behaviour in response to predator cues [47, 53]. The thorough activity and low docility of pikas from GN (lowest altitude) may facilitate them to obtain sufficient energy to satisfy greater energy requirements for reproduction.

Our findings indicate that differences in environmental conditions resulted in specific associations between behavioural traits of plateau pikas. These results indicate that behavioural syndromes could be adaptive, such that associations between behaviour may vary substantially in accordance with prevailing environmental conditions. Activity and docility were negatively associated in pikas from high- and medium-altitude sites. Correlations between these traits may corroborate the POLS hypothesis [13]. In the Tibetan Plateau, an increase in altitude is associated with a decrease in temperature and food resources [54]. Environmental or animal status may affect relationships between behavioural and physiological traits [55]. Negative feedback mechanisms (e.g. asset protection hypotheses) may predominate at low resource levels and decouple such correlations. By contrast, positive feedback mechanisms between animal states and different behaviour (e.g. starvation avoidance or state-dependent safety hypotheses) at intermediate levels of resource availability can produce behavioural syndromes [56]. Our study implied that the directions of such relationships could be affected by altitude. In high-altitude populations, negative associations between activity and docility may be adaptive and enable pikas to obtain additional food resources and evade predation. Pikas from lowest sites occupy higher vegetation cover and food availability, which can weaken or eliminate the correlations between activity and docility in pika populations [26].

We analysed behavioural differences among populations at the phenotypic level. As a result, we cannot rule out whether phenotypic differences reflect genetic differences or developmental plasticity [25]. Regardless of the cause of differences among populations, our results suggest that the divergence in behaviour traits across the altitudinal gradient may enable pikas to cope with different local environments [13, 17]. Reciprocal translocation or common-garden experiments would help to determine if personality differences reflect genetic differences or developmental plasticity [57–59].

We were unable to conclusively identify the precise divergences in the personality of pika populations distributed at higher altitudes, given that we focused on the traits of pika populations distributed within a limited altitude range. Nevertheless, our data reveal that the personality of pikas differs across altitudinal sites. Our study also provides valuable information regarding the effects of ecological conditions on the behavioural traits of animals at the population levels [13]. Although numerous studies have documented variation in life-history traits and phenotypes in response to global climate change [4], only a few have shown adaptations of animal personality to global warming [26, 60]. Increases in altitude are associated with decreases in temperature, and changes in environments may reflect how animals will respond to future climate change [3]. Further studies illuminating the mechanisms underlying the personality differences among populations are necessary to improve our understanding of animal responses to future climate change [61–63].

## Conclusions

Plateau pikas exhibit significant differences in activity and docility along the altitudinal gradient, supporting the POLS hypothesis. Future studies are needed to explore the mechanism and ecological consequences of personality divergences at large spatial-temporal scales and combine the animal personality with global climate changes.

## Data Availability

The dataset supporting the conclusions of this article are included in 10.6084/m9.figshare.7357313.v1.

## Abbreviations

GN: Guinan
MQ: Maqin
POLS: pace-of-life syndrome
ZK: Zeku
